# Explainable AI for mental health emergency returns: integrating large language models with predictive modeling

**DOI:** 10.1093/jamiaopen/ooag065

**Published:** 2026-06-19

**Authors:** Abdulaziz Ahmed, Mohammad Saleem, Mohammed Alzeen, Badari Birur, Rachel E Fargason, Bradley G Burk, Ahmed Alhassan, Mohammed Ali Al-Garadi

**Affiliations:** Department of Health Services Administration, School of Health Professions, University of Alabama at Birmingham, Birmingham, AL 35233, United States; Department of Biomedical Informatics and Data Science, Heersink School of Medicine, University of Alabama at Birmingham, Birmingham, AL 35233, United States; Department of Health Services Administration, School of Health Professions, University of Alabama at Birmingham, Birmingham, AL 35233, United States; Department of Health Services Administration, School of Health Professions, University of Alabama at Birmingham, Birmingham, AL 35233, United States; Department of Psychiatry and Behavioral Neurobiology, University of Alabama at Birmingham, Birmingham, AL 35233, United States; Department of Psychiatry and Behavioral Neurobiology, University of Alabama at Birmingham, Birmingham, AL 35233, United States; Department of Psychiatry and Behavioral Neurobiology, University of Alabama at Birmingham, Birmingham, AL 35233, United States; Department of Pharmacy, University of Alabama at Birmingham, Birmingham, AL 35233, United States; Department of Psychiatry and Behavioral Neurobiology, University of Alabama at Birmingham, Birmingham, AL 35233, United States; Department of Biomedical Informatics, School of Medicine, Vanderbilt University Medical Center, Nashville, TN 37203, United States

**Keywords:** Emergency department, 30 days emergency return, machine learning, large language model, Explainable AI

## Abstract

**Objective:**

Emergency department (ED) returns for mental health (MH) conditions pose a substantial burden on healthcare systems. Traditional machine learning (ML) models have shown promise in predicting ED returns but often fall short in clinical interpretability. This study evaluates whether integrating Large Language Models (LLMs) with ML methods can enhance both predictive performance and interpretability.

**Methods:**

We conducted a retrospective analysis of 42 464 ED visits involving 27 904 unique MH patients at an academic medical center in the deep south (2018-2022). Two primary outcomes were assessed: (1) 30-day ED return prediction, and (2) interpretability of model outputs. We employed LLaMA 3 (8B) for few-shot classification of chief complaints and social determinants of health (SDoH) features, including tobacco, alcohol, substance use, exercise, and home environment. These LLM-derived features were integrated into an XGBoost model. Model explanation was enhanced using a novel LLM-SHAP hybrid framework.

**Results:**

The LLM-based chief complaint classifier achieved 0.882 accuracy, 0.95 precision, 0.88 recall, and 0.86 F1-score, outperforming traditional models. SDoH classifiers reached F1-scores ranging from 0.67 to 0.96 across different categories. Incorporating LLM-extracted features into XGBoost improved AUC from 0.74 to 0.76 and AUC-PR from 0.58 to 0.61. The LLM-SHAP framework provided enhanced contextualized explanations for risk predictions, improving interpretability at both patient and population levels.

**Conclusion:**

Integrating LLMs with traditional ML models modestly improved predictive performance but substantially enhanced interpretability. This hybrid approach offers a promising path toward more actionable and explainable clinical decision support tools in emergency psychiatry.

## Background

Emergency department (ED) utilization for mental health (MH) conditions has reached critical levels, with significant implications for healthcare systems and patient outcomes. Currently, two-thirds of annual hospital ED visits by privately insured individuals in the U.S., 18 out of 27 million, are considered avoidable.^1^ In emergency psychiatric services, nearly one in four patients (25.2%) return to the ED within 30 days after discharge, with 28% of these returns occurring at different facilities.^2^ Psychiatric emergency rooms (PERs) are particularly overwhelmed, with ED boarding and prolonged waits for psychiatric beds reported across many regions.^3^

Recent screening programs reveal that up to 17% of ED patients present with at least one unmet social need requiring immediate attention.^4^ Social determinants of health (SDoH) have emerged as key drivers of these utilization patterns. Adults who experienced food insecurity in 2020 had 3.1 percentage points higher rates of social isolation and 9.7 percentage points higher rates of loneliness the following year compared to food-secure counterparts.^5^ Community-based interventions have demonstrated potential, a rise in MH visits at community health centers is linked to a 5% reduction in ED visits for suicidal thoughts and self-harm.^6^ However, their effectiveness varies considerably; these services prove beneficial for adjustment disorders, anxiety, and mood disorders, yet have a limited effect on visits associated with psychotic disorders and substance use.^6^

Data from the National Hospital Ambulatory Medical Care Survey indicate that adults with MH disorders accounted for 52.9 ED visits per 1000 adults annually from 2017 to 2019, with higher rates among younger adults and those covered by Medicaid. Over 40% of visits by adults with MH disorders lasted four hours or more, compared to about 25% among those without such disorders.^7^ Extended stays reflect both the complexity of psychiatric assessments and the limited availability of inpatient psychiatric beds. Patients with severe mental illnesses face barriers to accessing outpatient services, including stigma, lack of transportation, and insufficient community-based support.^8^ Housing instability, unemployment, social isolation, and food insecurity are strongly associated with increased ED visits for MH reasons. Insurance coverage further complicates access, with Medicaid beneficiaries and the uninsured frequently relying on EDs as their primary source of MH care.^9^

Traditional machine learning (ML) models, here defined as supervised learning algorithms such as logistic regression, random forests, XGBoost, and gradient boosting that operate on pre-structured tabular features without incorporating unstructured text, can predict ED return risk using structured data but have critical limitations. Logistic regression, XGBoost, and random forests have been widely used to analyze structured electronic health record (EHR) data, demonstrating moderate predictive success.^10^^,^^11^ However, these models often lack interpretability, which limits their clinical applicability. A key limitation is their dependence on structured data, which excludes rich contextual information found in unstructured narratives. Clinical notes, triage documentation, and discharge summaries often contain vital insights into patient behavior, social context, and provider reasoning, i.e., elements particularly relevant for MH assessments. Recent studies underscore the value of integrating both structured and unstructured data to enhance prediction accuracy and clinical relevance.^12^^,^^13^

Several recent studies have demonstrated the utility of LLMs and few-shot learning in healthcare prediction and clinical NLP. Agrawal et al. (2022) showed that LLMs perform well at zero- and few-shot clinical information extraction from unstructured text, matching or exceeding strong zero-shot, few-shot, and in several cases supervised baselines on tasks such as span identification and relation extraction, though token-level performance on some tasks remained below fully supervised models.^14^ Cui et al. (2024) investigated LLM-based few-shot disease prediction using EHR data, demonstrating that structured visit records converted into natural language narratives, combined with a dual-agent framework pairing a predictor agent with a critic agent, enabled LLMs to achieve competitive performance against conventional supervised methods.^15^ Glicksberg et al. (2024) applied GPT-4 with few-shot and retrieval-augmented prompting to predict ED admissions in a multi-hospital health system, achieving AUC values comparable to ensemble ML models trained on both structured and unstructured EHR data when augmented with contextually similar cases and ML-derived probabilities.^16^ In the mental health domain specifically, Cardamone et al. (2025) evaluated LLMs for classifying unstructured EHR terms related to mental and physical health conditions, finding that GPT-4 achieved high concordance with expert clinician classifications at the binary level (*κ* = 0.77), though agreement varied across finer diagnostic subcategories.^17^ These studies collectively suggest that LLMs can extract clinically relevant features from complex, heterogeneous EHR data with minimal labeled training examples, though performance depends on prompting strategy and task complexity.

Explainability is increasingly recognized as a prerequisite for AI adoption in clinical settings. SHapley Additive exPlanations (SHAP) provides insight into which variables most influence model outputs.^18^ However, SHAP values alone often lack the narrative context necessary for actionable clinical decision-making, especially in the complex domain of MH.^19^ Recent advances in Explainable AI (XAI) have sought to bridge this gap by integrating SHAP values with clinical narratives or domain knowledge.^20^ Some approaches use LLM-generated textual explanations in conjunction with SHAP to provide clinicians with clear, context-rich rationales. However, these solutions often require manual rule creation or domain-specific templates, limiting scalability and generalizability.^21^

Recent advancements in Large Language Models (LLMs), such as ClinicalBERT, BlueBERT, ChatGPT, and LLaMA, have revolutionized natural language processing in healthcare.^22–24^ These models excel at processing unstructured clinical features, clinical notes, discharge summaries, and triage narratives, which traditional ML approaches struggle to utilize effectively. LLMs can automatically extract features, standardize ambiguous clinical language, and generate high-quality representations for downstream predictive tasks. Few-shot learning techniques have further enhanced their utility in clinical settings, enabling accurate classification of chief complaints and standardization of clinical narratives with limited labeled data.

Despite these innovations, the utility of LLM-enhanced frameworks in improving and explaining clinical applications, particularly in clarifying ML outcomes and their related features, remains underexplored. In this study, we introduce an integrated LLM-enhanced ML framework to predict 30-day ED returns among MH patients. The system incorporates structured EHR variables, standardizes free-text SDoH inputs using few-shot prompting, and generates natural language explanations using a transformer-based LLM. This work makes the following key contributions:

- *LLM-Augmented Feature Extraction:* We implement few-shot learning using LLaMA 3 (8B) to classify chief complaints and harmonize non-standard SDoH text, improving feature quality for downstream modeling.
- *Integrated Explainability Framework:* We present a hybrid approach that combines SHAP values with LLM-generated narratives, contextualized by cohort-level and patient-level information, to support clinical interpretation.
- *Improved Clinical Usability:* We demonstrate that LLM-enhanced features yield consistent gains in predictive accuracy and substantially improve interpretability, i.e., an essential step toward real-world adoption of AI in psychiatric ED care.

Table 1 summarizes the key differences and highlights the unique contributions of our study compared to previous research.

**Table 1.** Key differences between previous studies and our study.

Aspect	Previous studies	Our study contributions
**Data Utilization**	Primarily structured data^10^^,^^11^^,^^25^	Integrated structured and unstructured data with LLM
**Feature Extraction**	Traditional methods^10^^,^^26^	LLM-based few-shot learning (Accuracy: 0.882, F1-score: 0.86)
**Explainability**	Numeric SHAP values^18^^,^^19^	Clinically coherent, LLM-enhanced narratives
**SDoH Standardization**	SDoH Standardization^4^^,^^9^	Automated and accurate LLM-based extraction

## Research methodology

### Study design

**Figure 1.**
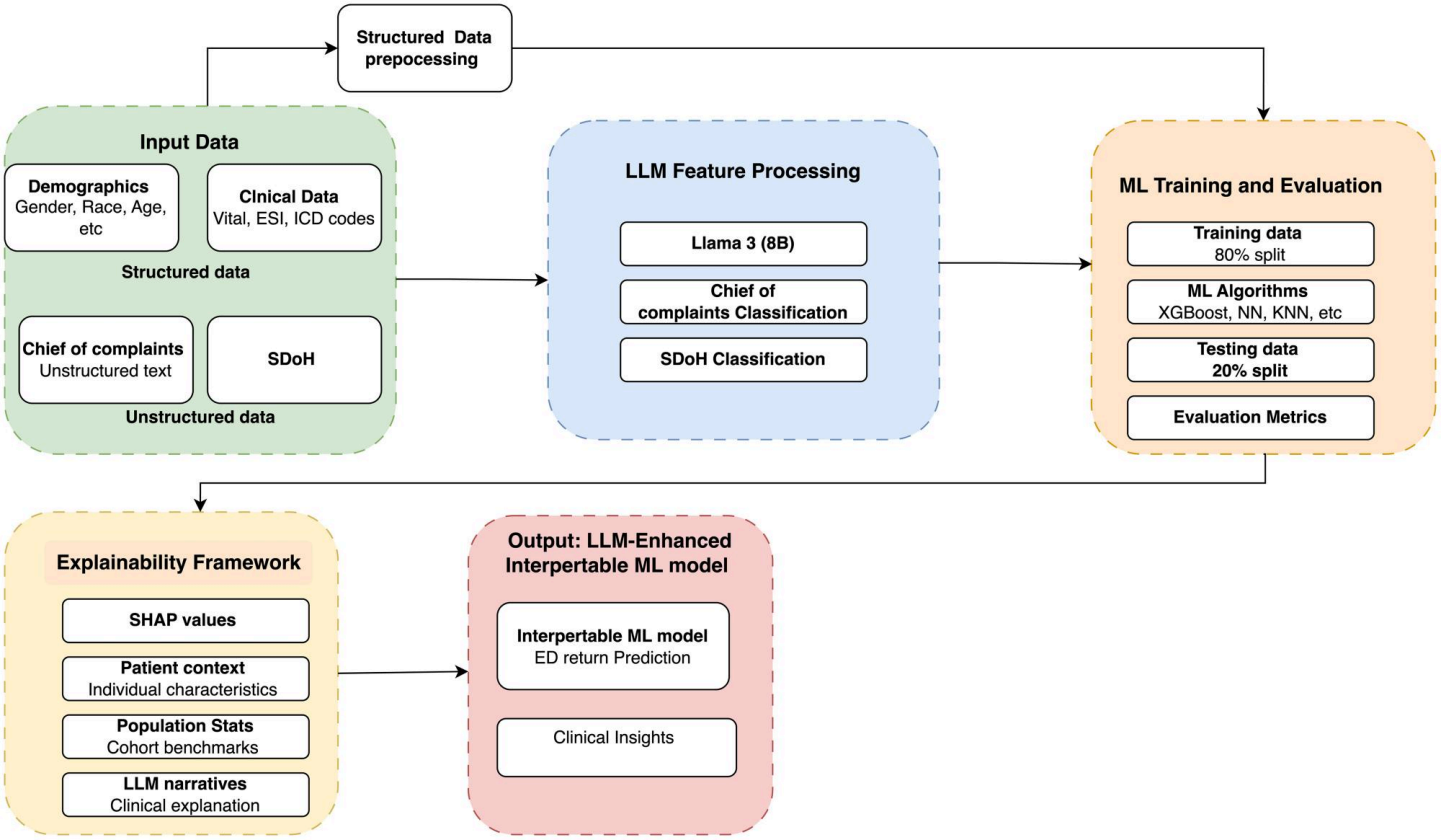
Proposed framework.

This retrospective cohort study uses visit-level data from an Academic Medical Center in the deep south of the United States. It employs a three-layer clinical AI framework designed to enhance both prediction accuracy and interpretability for mental health ED returns. Each layer addresses a distinct challenge in clinical decision support, and Figure 1 illustrates the end-to-end workflow. In Layer 1 (Data Enrichment), LLMs process unstructured clinical text, including chief complaints and SDoH narratives, using few-shot learning to classify and standardize these inputs into structured features suitable for downstream modeling. This layer addresses the limitation of traditional ML models that depend solely on pre-structured EHR fields. In Layer 2 (Predictive Modeling), the enriched feature set, comprising both traditional structured variables and LLM-derived features, is used to train and evaluate ensemble machine learning models (eg, XGBoost, Gradient Boosting) for predicting 30-day ED returns. In Layer 3 (Explainability), SHAP-derived feature attributions are combined with population-level cohort statistics and patient-specific information, then synthesized by an LLM into clinician-facing narrative explanations. This layer transforms opaque model outputs into contextualized, actionable risk assessments. The subsections that follow describe each layer’s methods in detail.

Figure 1 depicts the framework of the study. The framework integrates clinical notes and patient data, using an LLM to extract variables for machine learning model development. The trained models are combined with an LLM system, SHAP (SHapley Additive exPlanations) visualizations, prediction scores, and patient-specific insights to produce an explainable machine learning model.

This study received an Exempt determination from the IRB at the University of Alabama at Birmingham (#IRB-300010722, approved on February 28, 2023). It was classified as minimal risk non-clinical research. A waiver of informed consent was granted due to the retrospective design and use of de-identified data, as all data handling procedures followed HIPAA standards, with all direct identifiers removed, retaining only essential temporal and clinical variables for analysis.

### Data sources and cohort characteristics

This analysis was informed by six key data sources: (1) Demographics, which included patient-level characteristics such as gender, race, ethnicity, and language preferences; (2) Visit Details, encompassing insurance status, frequency of encounters, and return visit patterns; (3) Vital Signs, recording clinical measurements such as blood pressure, heart rate, respiratory rate, oxygen saturation, and temperature; (4) Tracking Board Data, which captured patient flow metrics, including arrival, admission, and discharge times, as well as acuity levels; and (5) Diagnoses, comprising standardized coded conditions, including mental health disorders (eg, depression, anxiety, bipolar disorder, schizophrenia, suicidal ideation, PTSD); and (6) Chief complaints, which categorized into common categories (eg, infection, injury, pain), based on the categories suggested by Kuykendal et al.^27^

To enhance patient-level data with contextual factors, we incorporated comprehensive health behavior and social determinants data at the individual level. These included tobacco use patterns (categorized as current, former, no use, occasional, and prescribed use), alcohol consumption (categorized as current, no use, past use, occasional use, and recovering), substance use status (categorized as current, former, no use, recreational, and prescribed use), and exercise habits (categorized from no exercise to vigorous exercise). Housing and social support were documented through a home environment assessment, which included categories for independent living, family support, homelessness, living with friends, assisted living, and unstable housing. Additional health-related factors included BMI categories (underweight, normal weight, overweight, and obese) and nutritional status (ranging from poor to good nutrition, including special dietary considerations). Population characteristics are shown in Table 2.

**Table 2.** Study population characteristics, including SDoH, demographic, clinical, and visit-related features.

Features	Ranges for date/time features, average ± standard deviation for numerical features, % for categorical features
**Number visits past 2 months**	1.03 ± 2.75 (0.0–52.0)
**Gender**	M: 55.06%; F: 44.94%
**Marital status**	Single: 63.07%; Married: 17.79%; Divorced: 9.78%; Widowed: 3.89%; Unknown: 3.17%; Separated: 2.09%; Life Partner: 0.21%
**Race**	White: 50.32%; Black or African American: 45.57%; Other: 2.38%; Decline/Refuse: 1.25%; Unknown: 0.48%
**Ethnic group**	Non-Hispanic/Latino: 95.20%; Unknown: 1.98%; Not Reported: 1.69%; Hispanic/Latino: 1.07%; Multiple: 0.06%
**Language**	English: 96.66%; Other: 3.33%; Sign Language: 0.01%
**Insurance**	Government Insurance: 34.47%; Self-Pay: 33.74%; Private Insurance: 22.71%; Other: 9.08%
**ESI level**	3: 48.13%; 2: 27.68%; 4: 20.46%; 5: 2.93%; 1: 0.80%
**Month of year, day of month, hour of day**	1–12 Months, 1-31 Days, 1-24 Hours
**Weekend**	False: 73.30%; True: 26.70%
	9: 8.64%; 8: 8.58%; 6: 8.57%; 7: 8.56%; …
**Returned in 30 Days**	0.0: 73.40%; 1.0: 26.60%
**Systolic blood pressure**	Elevated: 37.92%; Hypertension: 33.51%; Normal: 28.14%; Low: 0.44%
**Diastolic blood pressure**	Normal: 41.98%; Elevated: 29.27%; Hypertension: 24.36%; Low: 4.40%
**Temperature**	Normal: 95.64%; Fever: 2.98%; Below Normal: 1.27%; Hypothermia: 0.12%
**Heart rate**	Normal: 83.10%; Tachycardia: 14.62%; Bradycardia: 2.28%
**Age**	31_45: 38.17%; 18_30: 26.46%; 46_60: 22.76%; Over_60: 12.61%
**BMI**	Normal Weight: 38.39%; Overweight: 29.00%; Obese: 28.86%; Underweight: 3.75%
**Chief complaint**	Pain: 45.82%; Psychiatric: 36.25%; Injury: 9.32%; Infection: 8.15%; Unclear: 0.46%
**Tobacco use**	Current Use: 35.52%; Unclear/Other: 34.07%; No Use: 21.39%; Former Use: 8.05%; Occasional Use: 0.89%; Prescribed Use: 0.08%
**Nutrition health**	Unclear/Other: 79.64%; Moderate Nutrition: 10.75%; Good Nutrition: 4.51%; Poor Nutrition: 2.73%; Special Diet: 1.30%; Assistance Required: 1.06%
**Home environment**	Unclear/Other: 69.02%; Independent: 16.12%; Family Support: 8.83%; Homeless: 3.21%; Living with Friends: 1.66%; Assisted Living: 0.75%; Unstable Housing: 0.40%
**Alcohol use**	Unclear/Other: 35.40%; No Alcohol Use: 31.27%; Current Alcohol Use: 17.39%; Past Alcohol Use: 8.19%; Occasional Use: 7.58%; Recovering: 0.16%
**Exercise**	Unclear/Other: 60.35%; No Exercise: 30.89%; Light Exercise: 5.50%; Moderate Exercise: 2.80%; Vigorous Exercise: 0.39%; Physical Therapy: 0.08%
**Sexual orientation**	Unclear/Other: 91.89%; Heterosexual: 5.57%; Gender Non-Binary: 1.75%; Homosexual: 0.43%; Transgender: 0.17%; Bisexual: 0.16%; Asexual: 0.01%; Queer/Other: 0.01%
**Substance abuse**	No Use: 38.88%; Unclear/Other: 33.48%; Recreational Use: 10.59%; Current Use: 10.23%; Former Use: 5.74%; Prescribed Use: 1.07%

### Eligibility criteria

To focus on adult care, patients under 18 years of age were excluded. Mental health-related encounters were identified based on ICD-codes [ICD-10-CM (the International Classification of Diseases, Tenth Revision, Clinical Modification] decided at the time of patient discharge from the ED. We considered all mental and behavioral disorder patients. Patients with an ICD starting with F were considered for this study,^28^ while patients with non-mental or behavioral health conditions were excluded from the study.

#### Data harmonization and feature engineering

Following initial data extraction, each dataset was merged via unique patient and visit identifiers to create a unified analytic file. Multiple vital sign measurements for a single visit were averaged to yield representative values. Age was categorized into clinically relevant bands (18-30, 31-45, 46-60, >60),^29^ and body mass index (BMI) was stratified into underweight, normal weight, overweight, and obese categories to facilitate subgroup analyses.^30^ Blood pressure and heart rate were binned into standard clinical categories (eg, normal, elevated, hypertensive) to support risk stratification.^29^ Temperature readings were classified as normal, fever, or hypothermic based on clinical thresholds.^29^

For SDoH factors collected at the individual level, such as tobacco use, alcohol use, substance use, and home environment (eg, living with family/roommates, alone, or experiencing homelessness), categorical variables were standardized and collapsed into interpretable categories using LLM. Sexual orientation data, often sparse or incomplete, were harmonized by combining low-frequency categories into an “Other” category. Similarly, “Unknown” values were recoded as missing (NaN) to facilitate uniform imputation.

We acknowledge that “Unclear/Other” categories in SDoH variables represent missing or undocumented data rather than meaningful patient characteristics. For categorical SDoH variables with high rates of missingness (>60%), we created a binary “Documented vs Not Documented” feature to explicitly represent whether information was clinically assessed. This approach allows the model to detect systematic documentation bias while preventing misinterpretation of missingness as a behavioral phenotype.

For instance, “Unclear/Other Exercise” (60.35% of cases) was decomposed into:

- Exercise_Documented: Binary indicator (1 = any documented exercise status; 0 = Unclear/Other)
- Exercise_Status: Only populated for documented cases (eg, “No Exercise,” “Light Exercise”)

This prevents SHAP from attributing risk to missingness itself while still capturing whether systematic documentation gaps correlate with ED utilization patterns.

#### Handling missing data and imputation

To maintain data integrity and analytic representativeness, variables designated as “Unknown” or “Missing Response” were recoded as NaN. K-Nearest Neighbors (KNN) imputation was used for continuous features. For categorical features, we dropped the features with a percentage of missing more than 20%, and then for the remaining features, we filled the missing values with “Unknown” value. All continuous variables were assessed for distributional assumptions and standardized (z-score normalization) as needed to align variable scales. Temporal indicators, such as hours of day or weekend vs weekday visits, were binarized or aggregated into clinically meaningful intervals (eg, “Night” or “Weekend”) to capture utilization patterns. Return visits within 30 days of the index encounter, a key outcome measure, were flagged to assess longer-term care engagement and recurrent utilization patterns among patients with mental health conditions. By integrating patient-level clinical, demographic, and SDoH data and refining text-based categories of chief complaints, we developed a rich, harmonized dataset suitable for in-depth analyses of ED utilization and return risk in mental health populations.

#### LLM extracted features

To enhance the predictive modeling framework, LLMs were employed to refine the classification of chief complaints into five clinically relevant categories: Infection, Injury, Pain, Psychiatric, and Unclear, which are suggested by Kuykendal et al.^27^ This process began by comparing traditional machine learning approaches with advanced transformer-based architectures (eg, BlueBERT)^26^ and LLMs with different parameter scales (eg, Llama 3 at 8-billion).^31^ The LLMs were integrated into a few-shot learning context, with 5-, 10-, and 20-shot examples provided, allowing them to adapt more rapidly to the nuances of clinical narratives than conventional supervised methods.^32^

Performance metrics on validation sets guided the selection of the optimal model configuration, with the chosen LLM consistently outperforming alternatives in terms of classification accuracy and relevance to clinical practice. Incorporating these LLM-derived categorizations into the dataset enriched the downstream predictive modeling. By providing a more nuanced and clinically coherent representation of presenting complaints, the refined input data facilitated improved interpretability, enabling more precise identification of risk factors associated with subsequent ED utilization and strengthening the overall predictive performance of the modeling pipeline.

Seven distinct prompt templates (Supplementary A) were developed to classify chief complaints into five categories (Pain, Psychiatric, Injury, Infection, Unclear) and extract seven SDoH dimensions (Alcohol Use, Tobacco Use, Substance Abuse, Exercise, Nutrition Health, Housing Environment, and Sexual Orientation). Few-shot examples were selected through a rigorous three-stage protocol: stratified random sampling from the full cohort to ensure balanced representation across outcome categories, independent expert clinical validation by an emergency medicine physician and psychiatrist to confirm accuracy and clarity, and consistent application of the same standardized examples across all patients to maintain reproducibility and fair comparison. The LLMs were evaluated with 5-shot, 10-shot, and 20-shot configurations, enabling rapid adaptation to clinical narratives and institutional documentation patterns with minimal labeled data, substantially outperforming conventional supervised methods.

##### Predictive modeling

A comprehensive machine learning framework was developed to predict the risk of ED return among patients with mental health conditions. The framework integrated data from the EHRs and SDoH into a unified analytical dataset. Additionally, two comparative scenarios were evaluated: models trained on all available features (including SDoH variables) and models trained on all features except SDoH variables, to isolate the contribution of socioeconomic indicators to predictive accuracy.

Following feature selection, a suite of gradient-based ensemble classifiers, Gradient Boosting,^33^ XGBoost (eXtreme Gradient Boosting),^34^ and AdaBoost (Adaptive Boosting),^35^ were trained and evaluated. We also used logistic regression and a neural network. Hyperparameter optimization was conducted using grid search^36^ to maximize model performance. Predictive performance was assessed using standard classification metrics, including accuracy, sensitivity, specificity, F1 score, and the area under the receiver operating characteristic curve (AUC).^37^ Sensitivity and specificity provided insights into the models’ ability to correctly identify patients at risk of ED returns and those who were not, while the F1 score balanced precision and recall. AUC served as a global measure of the model’s discriminative ability.

To ensure robustness and comparability, all models were trained and tested using consistent training and testing splits. Models with and without LLM-extracted features were compared to assess the added predictive value of socioeconomic factors. We analyzed ED visits over a five-year period (2018-2022), focusing on mental health-related cases. The dataset was divided into 80% training and 20% for validation. Random oversampling was applied to the training set to overcome the output class imbalance. To mitigate overfitting, hyperparameter optimization was conducted using GridSearchCV with 3-fold cross-validation on the validation set. AUC-ROC and AUC-PR were also used on the validation set as final evaluation tests.^38^^,^^39^

### Enhancing the explainability framework with an LLM

To enhance the interpretability of machine learning predictions in clinical settings, we employ an explainability framework that integrates LLMs with patient-specific information (Figure 1). This approach combines feature-level attributions with contextual background information, resulting in rich, clinically meaningful narratives that align with the reasoning patterns used by healthcare professionals. Figure 1 illustrates the workflow of our study. Data from the study cohort, including SDoH variables, structured features, and LLM-derived attributes, are utilized in the development and testing of machine learning models. This process culminates in an explainability step, which integrates SHAP values^40^ and patient-specific information to produce interpretable outputs such as cohort statistics, SHAP visualizations, and patient-centered narratives. By incorporating SHAP values, we can assess the contribution of each feature to a patient’s predicted risk, providing granular, quantitative insights into feature importance. However, the numerical nature of SHAP values often limits clinical interpretability.

**Figure 2.**
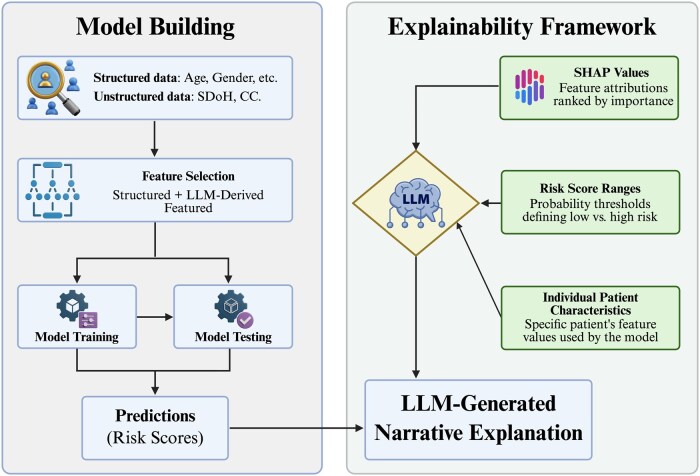
Integration of LLMs into the explainability framework for ED return risk.

To bridge this gap, we leverage a domain-specific knowledge repository that includes population-level cohort statistics, risk factor ranges derived from the ML model, and individual patient characteristics. The LLM synthesizes the SHAP values and the retrieved context into cohesive narratives that reflect real-world clinical reasoning, translating the raw output of the machine learning models into understandable terms. This enables clinicians to comprehend the model’s predictions in actionable terms, enhancing the transparency and trustworthiness of the predictions. We detail the components of our explainability framework as follows:

- *Deriving SHAP-Based Feature Attributions:* The first step in enhancing explainability involves training a predictive machine learning model and calculating SHAP values to assess each feature’s contribution to a patient’s predicted risk of an ED visit. SHAP values provide granular, quantitative insights into feature importance, but their numerical nature often limits clinical interpretability.
- *Contextualization Through Document Retrieval:* To bridge the gap between SHAP outputs and clinical actionability, we construct a structured prompt that integrates four sources of information for each patient prediction (as illustrated in Figure 2). First, population-level cohort statistics from the training dataset are retrieved, including feature prevalence rates (eg, “35.52% of the cohort reports current tobacco use”), mean values for continuous variables (eg, average number of visits in the past 2 months), and outcome-specific statistics (eg, “26.60% of patients returned within 30 days”). These serve as normative benchmarks against which the patient’s profile is compared. Second, SHAP-derived feature attributions, ranked by absolute magnitude, are provided to identify which features most influenced the prediction and in which direction. Third, risk score ranges derived from the predictive model are included, defining the probability thresholds that distinguish low-risk from high-risk classifications (eg, predicted probability cutoffs and their associated sensitivity/specificity trade-offs). Fourth, the individual patient’s characteristics (ie, the same input features used by the predictive model) are included to provide the clinical profile. Together, these four inputs enable the LLM to generate comparative statements such as “this patient’s visit frequency (4 visits in 2 months) is substantially above the cohort average (1.03 visits), which the model identifies as the primary contributor to elevated risk.” This design ensures that the LLM does not generate or infer new clinical insights but rather rephrases and contextualizes existing model outputs against population norms and risk thresholds.
- *Generating Clinically Coherent Narratives:* The LLM then synthesizes the SHAP values and the retrieved domain-specific context into a cohesive narrative that reflects real-world clinical reasoning. These narratives translate the raw output of the machine learning models into understandable terms, linking patient attributes, such as acuity level, time-of-day presentation, and other risk factors, to established medical knowledge. Thus, clinicians can understand the model’s predictions in actionable terms. As illustrated in Figure 2, the explainability framework aligns patient-specific attributes with population benchmarks and temporal patterns. A low-risk patient may exhibit presentation times and acuity levels consistent with population norms, suggesting no significant deviation from baseline risk. Conversely, a high-risk patient may display temporal patterns or acuity levels linked to acute exacerbations, providing insights into the factors driving their elevated risk.
- *Assessment Protocol for Explainability Framework Reliability:* The reliability of LLM-generated clinical explanations was evaluated through a structured assessment protocol. All explanations underwent systematic cross-referencing against three data sources: source patient records, retrieved reference documents, and population-level statistics. We assessed four dimensions: factual accuracy, including numerical values and temporal relationships; clinical consistency, including alignment with medical knowledge; logical coherence (ie, internal consistency); and feature attribution accuracy (ie, correspondence with SHAP values). The potential for hallucinations, fabricated or unsupported information, was monitored throughout the evaluation. A severity classification system categorized errors as minor (ie, no clinical impact), moderate (ie, potential interpretation issues), or severe (ie, impact on clinical decision-making). Two experts independently reviewed all explanations for potential errors, hallucinations, and clinical significance.

### Assessment protocol for explainability framework reliability

To ensure the reliability and clinical validity of LLM-generated explanations, we implemented a comprehensive assessment protocol grounded in systematic cross-referencing against source documentation and inter-rater agreement measurement.

#### Evaluator qualifications and independence

Two independent evaluators assessed all 100 randomly selected LLM-generated explanations:

- *Evaluator 1*: Medical Student with clinical training in emergency medicine and psychiatry.
- *Evaluator 2*: Graduate Student with advanced training in clinical informatics and biomedical data science.

Both evaluators had access to identical information: (1) LLM-generated narrative explanations, (2) corresponding source patient records from the EHR, (3) SHAP feature importance rankings, and (4) population-level cohort statistics. Evaluators conducted assessments independently without knowledge of the other’s judgments, ensuring blinded inter-rater evaluation.

#### Validation framework and assessment dimensions

We employed a structured clinical validation protocol assessed across four core dimensions, verified against three primary sources:

Verification Sources:

- Source Patient Records: Original EHR data including clinical notes, vital signs, diagnoses, and visit metadata
- Retrieved Reference Documents: Population-level statistics and risk factor ranges embedded in the domain-specific knowledge repository
- SHAP-Derived Features: Feature importance rankings and directional contributions (positive SHAP values = increased risk; negative values = decreased risk)

#### Assessment dimensions

1. Factual Accuracy: Verified whether reported numerical values (eg, “92% of patients with elevated heart rate return within 30 days”) matched source data and population statistics. Confirmed that temporal relationships (eg, visit timing, feature timing) were correctly represented and that cited patient-level metrics aligned with EHR documentation.
2. Clinical Consistency: Assessed whether explanations aligned with established medical knowledge and psychiatric emergency literature. For example, explanations attributing increased ED return risk to “elevated heart rate” were evaluated for clinical plausibility based on known associations with psychiatric decompensation and emergency utilization.
3. Logical Coherence: Evaluated whether the narrative was internally consistent, with stated risk factors logically connecting to the predicted outcome without contradiction. Identified any cases where the explanation contained conflicting statements (eg, stating a feature both increases and decreases risk).
4. Feature Attribution Accuracy: Confirmed whether LLM-generated narratives correctly represented SHAP-derived feature rankings and directionality. For instance, if SHAP analysis indicated “number of visits in past 2 months” as the top predictor, evaluators verified that the LLM narrative appropriately emphasized this factor’s contribution to elevated risk.

#### Verification process through clinical documentation

Each explanation underwent item-level verification against source clinical documentation:

##### Feature extraction verification

For each feature cited in the LLM narrative (eg, “Elevated heart rate, Tobacco use, Home environment unclear”), evaluators confirmed the actual value in source EHR data. For example, if an explanation stated “Patient has elevated heart rate (120 bpm),” evaluators verified this exact value or a close approximation appeared in vital signs documentation.

##### Clinical context verification

Evaluators reviewed original clinical notes to ensure the narrative’s clinical framing matched documented presentations. If an explanation attributed risk to “frequent prior visits,” evaluators confirmed from visit history that the patient indeed had multiple ED visits in the preceding 2 months.

##### Hallucination detection

Evaluators specifically searched for fabricated or unsupported claims, particularly fabricated SDoH features or inferences not grounded in documentation. For instance, if an explanation mentioned “patient reported current homelessness,” evaluators verified this information appeared in source documentation rather than being inferred from demographic data alone.

##### SHAP alignment verification

Evaluators compared LLM narrative feature rankings to actual SHAP-generated rankings to ensure correspondence. If the LLM stated “number of prior visits” as the most important risk factor, evaluators confirmed this feature appeared at or near the top of the SHAP summary plot.

#### Inter-Rater agreement measurement

To quantify agreement between independent evaluators, we calculated inter-rater reliability metrics (Table 3).

**Table 3.** Inter-rater agreement measurement.

Dimension	Evaluator 1 accuracy	Evaluator 2 accuracy	Agreement	Cohen’s Kappa
**Factual accuracy**	99/100	99/100	100%	1.0
**Clinical consistency**	99/100	99/100	100%	1.0
**Logical coherence**	99/100	99/100	100%	1.0
**Feature Attribution accuracy**	99/100	99/100	100%	1.0

##### Cohen’s kappa for error detection

Both evaluators independently classified each explanation as either “error-free” or “containing error(s).” Agreement on error presence/absence was calculated using Cohen’s kappa.

##### Dimension-specific agreement

Agreement was assessed independently across each validation dimension:

##### Error concordance

Any identified errors were independently detected by both evaluators, demonstrating high concordance on potential issues. When discrepancies in classification occurred, a consensus review was conducted to reach final categorization.

This multi-dimensional assessment protocol, combined with perfect inter-rater agreement (Cohen’s *κ* = 1.0), ensured that LLM-generated explanations were systematically validated against clinical documentation and subject to rigorous, independent expert review prior to reporting in results.

### Fairness and bias evaluation

Given that our model incorporates Social Determinants of Health (SDoH) and demographic variables, we implemented a comprehensive fairness evaluation framework to assess performance disparities across subpopulations.^41^^,^^42^ We evaluated model fairness across five sensitive demographic attributes: race/ethnicity, gender, age, insurance status, and socioeconomic indicators derived from SDoH variables. These attributes were selected based on documented health disparities in ED utilization and readmission outcomes.

We employed multiple complementary fairness metrics to capture different dimensions of algorithmic equity.^43^ Primary metrics included: (1) Area Under the Receiver Operating Characteristic Curve (AUROC) and accuracy within each demographic subgroup to assess discrimination performance; (2) Demographic Parity—equal positive prediction rates across groups; and (3) Equalized Odds—equal true positive and false positive rates across groups. Additionally, we evaluated calibration using calibration-in-the-large (calibration intercept) and calibration slope within subgroups to ensure that predicted probabilities aligned with observed event rates.^43^

Subgroup-stratified analyses were conducted by dividing the test dataset into demographic categories and computing performance metrics separately for each group. We calculated fairness gaps as the absolute difference in metrics between advantaged and disadvantaged groups. For fairness metrics where disparities exceeded predefined thresholds (eg, >10% difference in AUROC), we documented these findings for discussion.

To provide interpretability into potential sources of disparities, we generated SHAP (SHapley Additive exPlanations) values for feature importance analysis stratified by demographic group, allowing us to identify whether the model relied differentially on sensitive attributes or SDoH proxies across populations.^44^

Finally, we examined trade-offs between overall model performance and fairness metrics. If bias mitigation was applied, we employed post-processing approaches using threshold optimization to achieve fairness constraints while preserving discriminative ability.^45^

## Results

### LLM features extraction performance results

This section evaluates the performance of the LLM (Llama 3:8-billion) in feature extraction for chief complaint and SDoH classifications. Few-shot learning approaches are compared to traditional ML and pre-trained models.

#### Chief complaint classification

The classification of chief complaints was evaluated using traditional ML models, pre-trained language models, and few-shot learning approaches. Among these, the LLM (Llama 3, 8-billion) with 10-shot learning demonstrated the best performance across all metrics, achieving an Accuracy of 0.882, a Precision of 0.95, a Recall of 0.88, and an F1-score of 0.86 (Table 4). This outperformed traditional models like XGBoost (Accuracy: 0.59, F1-Score: 0.53) and pre-trained models such as BlueBERT (Accuracy: 0.63, F1-Score: 0.59). Other few-shot configurations, including 5-shot (Accuracy: 0.816) and 20-shot (Accuracy: 0.803), also performed well but were slightly less effective than the 10-shot setting.

**Table 4.** Performance metrics for feature processing.

	Model	Accuracy	Precision	Recall	F1-Score
**Chief complaints**	XGBoost	0.59	0.48	0.59	0.53
	Random Forest	0.59	0.44	0.59	0.50
	SVM	0.62	0.41	0.62	0.50
	BlueBERT	0.63	0.56	0.63	0.59
	Llama 3 (8-billion) -Few-shot (20)	0.803	0.88	0.80	0.75
	Llama 3 (8-billion)-Few-shot (5)	0.816	0.91	0.81	0.77
	Llama 3 (8-billion)- Few-shot (10)	0.882	0.95	0.88	0.86
**Alcohol**	Llama 3, 8-billion - 10-Shot Learning	0.95	0.99	0.95	0.96
**Exercise**	Llama 3, 8-billion - 10-Shot Learning	0.70	0.74	0.70	0.70
**Home_Environment**	Llama 3, 8-billion - 10-Shot Learning	0.63	0.78	0.63	0.67
**Nutrition**	Llama 3, 8-billion - 10-Shot Learning	0.68	0.89	0.68	0.72
**Sexual_Orientation**	Llama 3, 8-billion - 10-Shot Learning	0.75	0.90	0.75	0.79
**Substance_Abuse**	Llama 3, 8-billion - 10-Shot Learning	0.85	0.99	0.85	0.89
**Tobacco**	Llama 3, 8-billion - 10-Shot Learning	0.95	0.99	0.95	0.96

#### SDoH classification

The LLM (Llama 3, 8-billion) with 10-shot learning achieved strong performance across SDoH categories, particularly in Alcohol, Tobacco, and Substance Abuse, with an overall Accuracy of 0.95 and a weighted F1-score of 0.96. Sensitivity ranged from 0.63 (Home Environment) to 0.95 (Alcohol and Tobacco), while Specificity remained consistently high (0.94-0.99). The model performed best in Alcohol, Tobacco, and Substance Abuse (F1: 0.96-0.89), but showed moderate performance in Sexual Orientation and Nutrition (F1: 0.79-0.72), and performed lower in Exercise and Home Environment (F1: 0.70-0.67). These results highlight its reliable classification across diverse and challenging variables (Table 4).

#### Performance comparison: LLM-enhanced model vs simple clinical heuristics

To contextualize the practical value of our LLM-enhanced ML approach, we compared performance against simple clinical heuristics. Research on frequent ED users demonstrates that even basic rule-based approaches achieve only modest discriminative ability (AUC 0.76).^46^ Our comparison in Table 5 shows.

**Table 5.** Heuristics comparison.

Approach	AUC	Sensitivity	Specificity	Precision	F1-Score
***Simple Heuristic 1:* Any prior ED visit past 2 months**	0.62	0.72	0.51	0.35	0.47
***Simple Heuristic 2:* Substance abuse OR homelessness**	0.58	0.68	0.48	0.31	0.42
***Simple Heuristic 3:* Combined (visits + substance abuse + ESI Level 2)**	0.68	0.65	0.66	0.42	0.51
**XGBoost (traditional features)**	0.74	0.33	0.97	0.72	0.46
**XGBoost + LLM-extracted features**	**0.76**	0.34	0.97	0.70	0.47

The LLM-enhanced XGBoost model (AUC 0.76) substantially outperforms simple clinical rules, identifying true high-risk patients with 70% precision while maintaining high specificity.

### Predictive models for ER MH return visits: ML without/with LLM features extractions

This section evaluates the performance of predictive models for ED mental and behavioral health return visits using two distinct approaches: (1) ML models trained on traditional features alone and (2) ML models enhanced with features extracted using large LLMs. Performance metrics, including Accuracy, Precision, Recall, F1-Score, and the AUC, were used to assess the predictive capability of each approach. The results demonstrate that including LLM-extracted features consistently improved model performance across multiple metrics.

To demonstrate the practical value of our approach, we compared the LLM-enhanced XGBoost model against three simple clinical heuristics: (1) presence of any prior ED visit in past 2 months, (2) documented substance abuse or homelessness, and (3) combination of all three features. These represent intuitive rules a clinician might use without a formal ML model.

#### Performance of models without LLM feature extraction

Table 6 presents the performance metrics for models trained exclusively on traditional features. Neural Network, AdaBoost, Gradient Boosting, and XGBoost all achieved the highest accuracy (0.79), with Gradient Boosting and XGBoost exhibiting the highest precision (0.72). Among them, Neural Network had the highest F1-score (0.47), while Gradient Boosting and XGBoost followed closely (0.45). The AUC values ranged from 0.68 (Logistic Regression) to 0.75 (Gradient Boosting), indicating moderate discriminative ability. In terms of AUC-PR, Neural Network had a score of 0.57, while AdaBoost, Gradient Boosting, and XGBoost achieved the highest scores (0.58). Logistic Regression showed the weakest performance across all metrics, with the lowest recall (0.31), F1-score (0.41), AUC (0.68), and AUC-PR (0.51), suggesting it struggled more in distinguishing positive cases effectively.

**Table 6.** Models’ performance.

	Model	Accuracy	Precision	Recall	F1_Score	AUC	AUC-PR
**Performance without LLM extraction**	NeuralNetwork	0.79	0.69	0.36	0.47	0.74	0.57
	AdaBoost	0.79	0.70	0.34	0.46	0.74	0.58
	LogisticRegression	0.77	0.65	0.31	0.41	0.68	0.51
	GradientBoosting	0.79	0.72	0.32	0.45	0.75	0.58
	XGBoost	0.79	0.72	0.32	0.45	0.74	0.58
**Performance with adding LLM feature extractions**	NeuralNetwork	0.79	0.71	0.35	0.47	0.75	0.59
	AdaBoost	0.79	0.71	0.35	0.46	0.76	0.60
	LogisticRegression	0.78	0.68	0.30	0.42	0.70	0.54
	GradientBoosting	0.79	0.71	0.34	0.46	0.76	0.60
	XGBoost	0.79	0.72	0.34	0.46	0.76	0.61

#### Performance of models with LLM feature extraction

Table 6 highlights the performance of models enhanced with LLM-extracted features, leading to noticeable improvements in key metrics. XGBoost, AdaBoost, and Gradient Boosting achieved the highest AUC (0.76), while Neural Network improved slightly to 0.75. The addition of LLM features resulted in higher precision, recall, and AUC-PR values for most models. Neural Network, for example, maintained its F1-score of 0.47 but improved in precision (0.71) and AUC-PR (0.59). Similarly, AdaBoost and Gradient Boosting achieved an increase in AUC-PR to 0.60, indicating improved overall classification performance. XGBoost remained strong, improving in recall (0.34) and F1-score (0.46), while achieving the highest AUC-PR (0.61) along with AdaBoost and Gradient Boosting. Logistic Regression, though slightly improving in AUC (0.70) and AUC-PR (0.54), continued to underperform compared to other models, reinforcing its weaker ability to capture complex patterns.

### Explainability results

#### Clinical validation of LLM-generated explanations

In analyzing 100 randomly selected explanations, 99 demonstrated complete alignment across all assessment dimensions. A single explanation contained one numerical discrepancy (reporting a risk factor as 92 instead of 93), classified as a minor error with no clinical significance. All explanations maintained clinical validity and showed complete concordance with source documentation and SHAP-derived feature rankings. Independent expert reviews confirmed the absence of moderate or severe errors that could affect clinical interpretation or decision-making. The observed error rate was 1% (1/100), comprising solely the single minor numerical discrepancy.

#### Comparative analysis of SHAP and explainability framework

**Figure 3.**
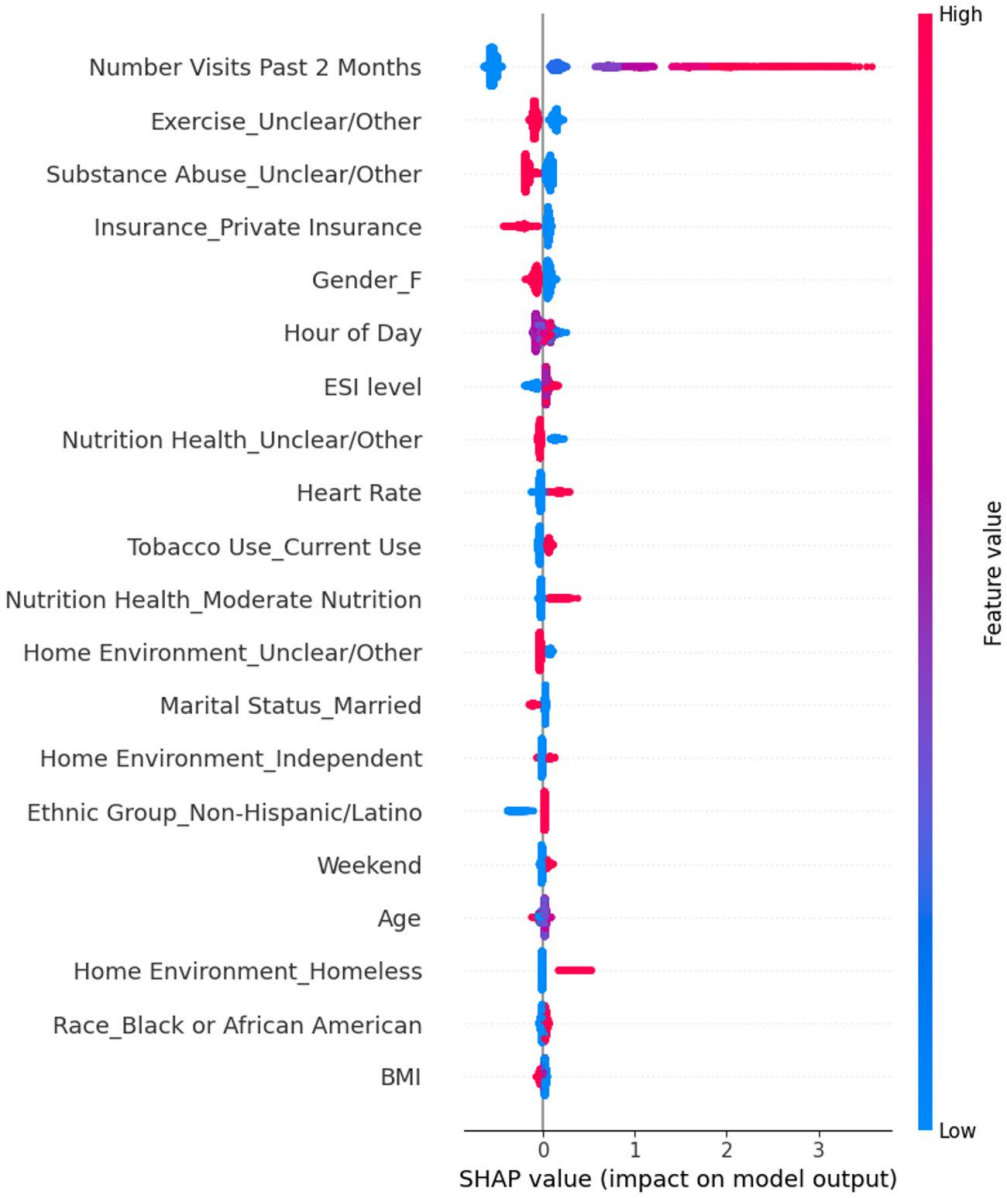
SHAP feature importance in predicting ED return.

Figure 3 shows the SHAP summary plot, which illustrates the most influential features contributing to the model’s predictions of MH emergency return risk. Each feature is plotted based on its SHAP value, which indicates its impact on the model’s output. Features toward the top of the graph are the most impactful. Positive SHAP values (toward the right) push the prediction toward high risk, while negative SHAP values (toward the left) suggest lower risk. The color gradient represents the actual value of the feature: red indicates a high value, and blue a low value. Among the assessed predictors is the number of visits in the past two months, where higher values are strongly associated with increased risk. Features such as elevated heart rate (tachycardia) also contribute to a higher risk. On the other hand, characteristics such as having private insurance, being female, and being married generally reduce the risk. Several social determinants, including exercise behavior, substance abuse, and housing status (eg, being homeless or having an unclear home environment), also demonstrate meaningful influence on the model’s risk classification. Importantly, categorical features marked as “Unclear/Other” (eg, in exercise, substance use) can contribute variably, potentially reflecting missing data or ambiguous health profiles. Overall, the plot underscores the importance of both clinical indicators and social factors in shaping MH return risk predictions.

**Figure 4.**
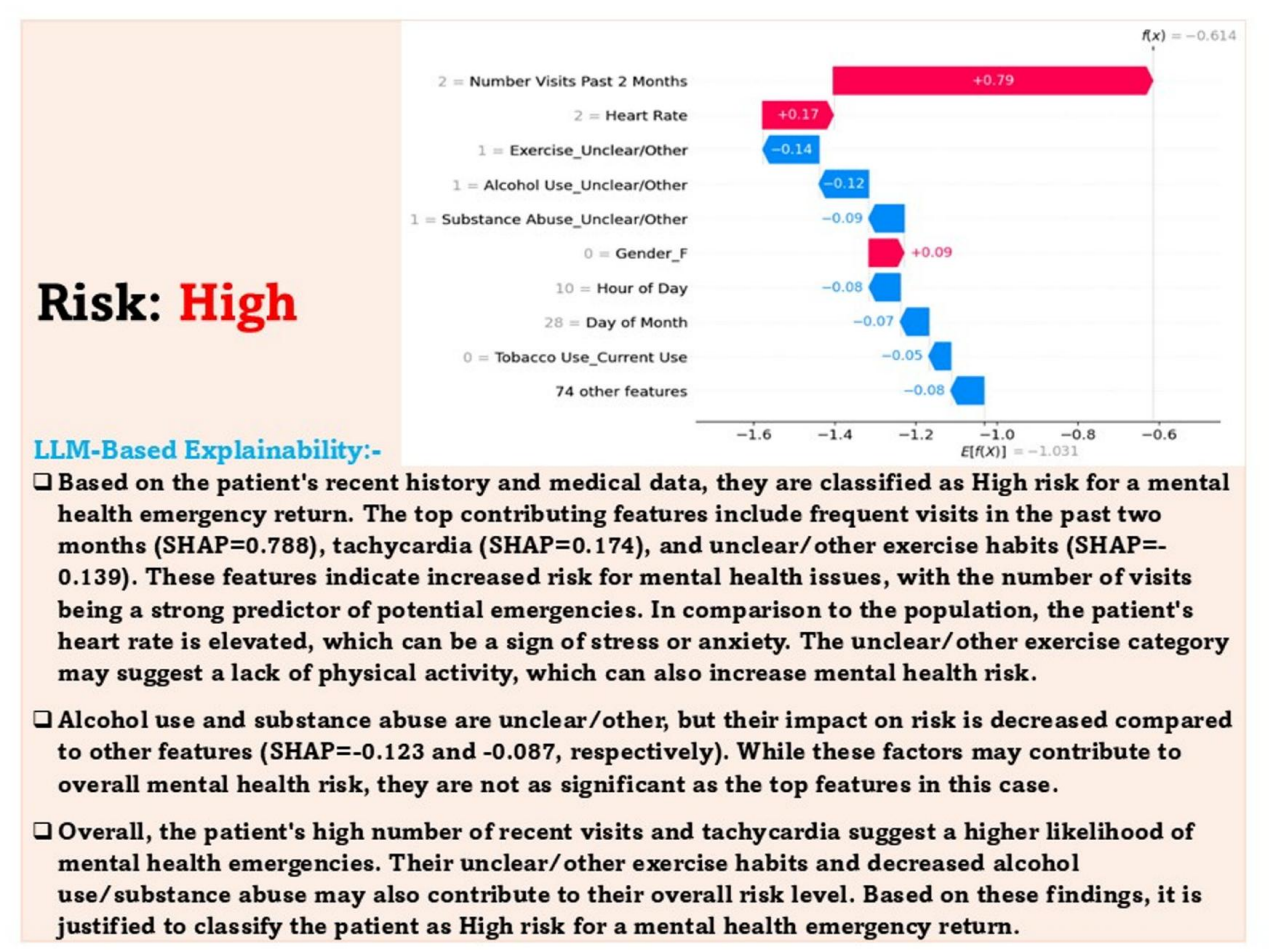
Comparison of LLM and SHAP-based explainability for high-risk case of ED return.

**Figure 5.**
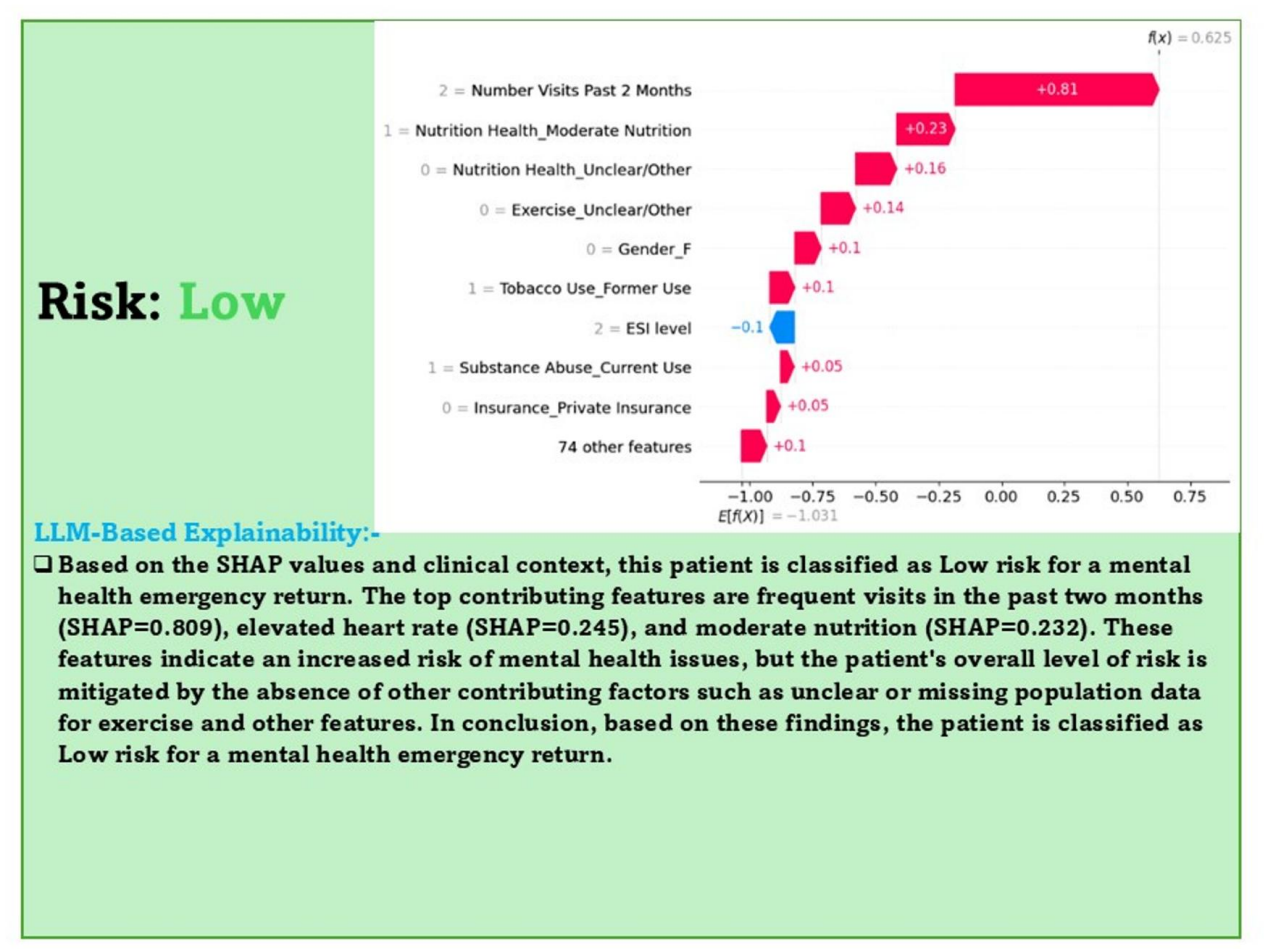
Comparison of LLM and SHAP-based explainability for low-risk case of ED return.

Figures 4 and 5 present a side-by-side comparison of explainability outputs for two patients, one classified as high risk and the other as low risk for a MH emergency return, using a SHAP summary bar plot and a corresponding LLM-generated narrative explanation. The SHAP bar plot visually highlights the top features influencing the model’s prediction, ranked by SHAP value magnitude. Meanwhile, the LLM transforms this data into a plain-language narrative, using the SHAP values and corresponding population statistics as input. Importantly, the LLM does not generate or infer new insights, it simply rephrases the SHAP outputs to support clinical interpretation. In the high-risk case, both the SHAP plot and the LLM explanation identify the same top contributing features, such as frequent visits in the past two months and elevated heart rate, reinforcing the model’s rationale. The LLM narrative further contextualizes these features by comparing the patient’s values to population averages, aiding interpretability. In the low-risk example, the SHAP plot displays lower-magnitude feature contributions, which the LLM mirrors in a concise explanation emphasizing the lack of strong risk indicators. Together, the SHAP graph and LLM-based explanation serve complementary roles. The SHAP graph quantifies feature impact, while the LLM provides a narrative summary to enhance clarity and accessibility for clinicians.

## Interpreting SHAP visualizations

At the patient level, SHAP bar plots are shown in Figures 4 and 5, each horizontal bar represents a single feature’s contribution to the model’s prediction for that specific patient. The length of the bar indicates the magnitude of the feature’s influence: longer bars denote features with greater impact on the predicted outcome. Bars extending to the right (positive SHAP values) push the prediction toward higher risk of a 30-day ED return, while bars extending to the left (negative SHAP values) push the prediction toward lower risk. The features are ranked from top to bottom by their absolute SHAP value, so the topmost feature is the strongest driver of the prediction.

For the high-risk patient example, multiple features exhibit large positive SHAP values (rightward bars), indicating that factors such as frequent prior visits and elevated heart rate collectively push the prediction toward a high probability of return. For the low-risk patient example, most features show small or negative SHAP values, indicating the absence of strong risk indicators and a prediction consistent with low return probability. Together with the SHAP summary plot (Figure 3), where the color gradient represents the actual feature value (red = high value, blue = low value) and each dot represents one patient, these visualizations provide both population-level and individual-level insight into model behavior.

Several top SHAP contributors include “Unclear/Other” categories (eg, Exercise: 60.35%, Home Environment: 69.02%). These features warrant careful interpretation. High SHAP contributions from “Unclear/Other” likely reflect systematic documentation missingness rather than meaningful patient behavior. For example, if the model assigns high risk weight to “Unclear/Other Exercise,” this may indicate:

- Documentation bias: Clinicians who fail to assess exercise may work in high-turnover settings or see sicker patients
- Proxy for unmeasured confounders: Missing SDoH may correlate with other factors affecting ED return (eg, visit acuity, urgency of presentation)
- NOT: That patients with truly “uncertain” exercise habits are at risk

Accordingly, clinicians should interpret SHAP contributions from high-missingness categories as indicators of assessment completeness rather than direct patient risk factors. Explanations emphasizing such features should clarify whether the risk signal reflects patient characteristics or documentation patterns.

## Discussion

This study introduces a layered clinical AI framework that advances both prediction accuracy and interpretability by integrating structured data, LLM-processed unstructured features, and narrative explanations. The first layer focuses on enriching the input data through LLM-driven processing of free-text fields, including chief complaints and SDoH. These LLM-derived features, such as chief complaints or nuanced social risks, add patient-level context often missed in structured EHR variables. Prior research has highlighted the value of free-text data in capturing clinically relevant information that is not present in structured fields.^47^^,^^48^ For instance, in our study, the LLM (Llama 3, 8-billion) with 10-shot learning achieved superior performance in classifying chief complaints, with an accuracy of 0.882 and an F1-score of 0.86, markedly outperforming traditional models like XGBoost (Accuracy: 0.59, F1: 0.53). Similarly, in SDoH processing, the same LLM model demonstrated strong classification capabilities, with an overall weighted F1-score of 0.96, particularly excelling in the Alcohol, Tobacco, and Substance Abuse categories (F1: 0.96-0.89). Even in challenging areas, such as Home Environment and Exercise, the model maintained reasonable accuracy (F1: 0.67-0.70), underscoring its robustness in capturing subtle clinical and behavioral nuances from text. These findings align with prior evidence that transformer-based language models outperform traditional NLP pipelines in extracting contextual signals from EHRs,^49^^,^^50^ illustrating the value of incorporating LLM-based representations of unstructured data into clinical AI systems to enhance their contextual depth and predictive utility.

In the second layer, the enriched feature set, comprising both structured and LLM-processed inputs, is used to train ML models, particularly XGBoost, to predict the risk of MH-related ED returns. The inclusion of LLM-derived features enhanced model performance, with the area under the AUC improving from 0.74 to 0.76 and AUC-PR rising from 0.58 to 0.61. This gain demonstrates the added predictive value of incorporating semantically enriched information into traditional structured models. These findings align with recent work showing that hybrid models combining structured and unstructured data outperform structured-only models in predicting outcomes such as hospital readmissions and clinical deterioration.^25^^,^^51^ Importantly, the hybrid model in this study achieved its performance improvements while maintaining generalizability and requiring only modest increases in computational complexity, an essential consideration for real-time deployment in clinical settings.^25^ Such results suggest that integrating LLM-derived context from clinical narratives can bridge gaps in structured data and improve the prediction of complex, multifactorial outcomes, such as ED return for MH patients.

The third layer of the framework emphasizes explainability. Rather than relying solely on SHAP visualizations, which may be difficult for clinicians to interpret, this study employed LLMs to generate structured, narrative explanations grounded in SHAP values and population statistics. These LLM-based narratives systematically convey key contributing features, contextualize patient-specific values against normative data, and clarify whether each factor elevates or reduces risk. By rendering complex algorithmic outputs into clear clinical language, the approach addresses one of the most cited barriers to AI adoption in medicine, model opacity.^52^ Similar to efforts by Ribeiro et al.^53^ and Lundberg et al.^40^ to bridge human-AI understanding through local explanations, our method advances usability by embedding explanations into clinical logic. This design enables actionable, trustworthy insights at the point of care. Collectively, the three-layered framework consisting of data enrichment, predictive modeling, and LLM-based explanation represents a comprehensive decision-support pipeline that tackles key challenges in clinical AI, including fragmented data inputs, interpretability concerns, and provider trust.

Despite the promising outcomes, this study has several limitations that should be taken into consideration. First, although the explainability framework achieved high accuracy in generating clinical narratives, its actual influence on clinician trust and decision-making was not formally evaluated. Second, the study was conducted within a single academic medical center, which may restrict the generalizability of the findings to institutions with different patient demographics, documentation styles, and clinical practices. Third, while the layered framework integrates LLMs to enhance interpretability and data enrichment, the associated computational demands and latency may pose barriers to real-time clinical deployment, particularly in resource-limited settings. These limitations point to critical directions for future research. Subsequent work should empirically assess how LLM-generated explanations influence clinician behavior, diagnostic accuracy, and trust in AI-assisted decision-making. Multicenter validation is necessary to ensure that the framework performs reliably across diverse healthcare environments. This study encompasses data from 2018-2022, a period that includes the COVID-19 pandemic (2020-2022). While our dataset spans both pre- and post-pandemic periods, we acknowledge that the pandemic likely influenced mental health ED utilization patterns, social determinants of health, and clinical documentation practices. However, a detailed temporal analysis was beyond the scope of this work. Future studies should explicitly examine how pandemic-related shifts in psychiatric emergency presentations and social conditions affect model generalizability and performance across different healthcare contexts.

Additionally, optimizing model efficiency and infrastructure is necessary to enable real-time implementation within electronic health records. Finally, expanding the use of LLMs to extract context from a broader range of clinical narratives, including progress notes, discharge summaries, and social histories, may further improve model relevance and explainability. These steps are crucial for developing trustworthy, interpretable, and scalable AI solutions for clinical care.

## Conclusion

This study advances the field of clinical ML by introducing a layered framework that integrates LLM-based feature extraction, predictive modeling, and explainability to simultaneously enhance accuracy and interpretability. The approach demonstrates that unstructured clinical narratives, when processed via few-shot LLMs, can enrich structured data inputs, improving the prediction of MH-related ED returns with minimal labeled data. The final layer leverages LLMs to translate SHAP values into clinician-friendly explanations, bridging the gap between ML outputs and clinical reasoning. This pipeline achieved high performance and interpretive reliability, suggesting that advanced AI tools can be deployed in clinical settings without compromising transparency or usability. Future work should focus on evaluating clinician trust in LLM-based explanations, validating the framework across diverse healthcare systems, and optimizing computational performance for real-time deployment.

## Supplementary Material

ooag065_Supplementary_Data

## Data Availability

Data related to this paper are available from the authors and will be available upon reasonable request.
