# Supplementary material for: Explainable AI for mental health emergency returns: integrating large language models with predictive modeling
Source: JAMIA Open. 2026 Jun 19;9(3):ooag065. doi: 10.1093/jamiaopen/ooag065 (PMC13278837; doi:10.1093/jamiaopen/ooag065)
Supplement: ooag065_Supplementary_Data [file ooag065_supplementary_data.docx]

# Supplementary A: Prompt Templates Used for LLM Classification

## S.1 Chief Complaint Classification Prompt

You are a medical classification assistant. Classify the following emergency department chief complaints into one of five categories: Pain, Psychiatric, Injury, Infection, or Unclear.
Examples:
1. Chief Complaint: "I can't stop vomiting." → Category: Infection
2. Chief Complaint: "Severe back pain after lifting boxes." → Category: Pain
3. Chief Complaint: "Hearing voices and suicidal thoughts." → Category: Psychiatric
4. Chief Complaint: "Cut hand with a kitchen knife." → Category: Injury
5. Chief Complaint: "Weakness for the past week, unknown cause." → Category: Unclear
Now classify the following:
Chief Complaint: "[NEW_CHIEF_COMPLAINT]" → Category:

## S.2 Alcohol Use Classification Prompt

You are a clinical classification assistant. Classify patient-reported alcohol use into one of the following categories:
- No Alcohol Use
- Current Alcohol Use
- Past Alcohol Use
- Occasional Use
- Recovering
- Unclear/Other
Examples:
1. Input: "No alcohol ever" → Category: No Alcohol Use
2. Input: "Drinks socially, rarely" → Category: Occasional Use
3. Input: "History of alcohol abuse, now sober" → Category: Recovering
4. Input: "Used to drink, quit 5 years ago" → Category: Past Alcohol Use
5. Input: "Drinks 3-4 times/week" → Category: Current Alcohol Use
6. Input: "No mention" → Category: Unclear/Other
Now classify:
Input: "[ALCOHOL_TEXT]" → Category:

## S.3 Nutrition Health Classification Prompt

You are a clinical assistant classifying nutrition-related responses. Use the following categories:
- Balanced Diet
- Unhealthy Diet
- Irregular Eating Habits
- Malnutrition Risk
- Unknown/Other
Examples:
1. Input: "Eats fast food every day" → Category: Unhealthy Diet
2. Input: "Three meals a day, includes vegetables" → Category: Balanced Diet
3. Input: "Sometimes skips meals" → Category: Irregular Eating Habits
4. Input: "Underweight and reports poor appetite" → Category: Malnutrition Risk
5. Input: "No data provided" → Category: Unknown/Other
Now classify:
Input: "[NUTRITION_TEXT]" → Category:

## S.4 Tobacco Use Classification Prompt

You are a clinical assistant. Classify tobacco use into one of the following:
- Never Smoked
- Current Smoker
- Former Smoker
- Occasional Smoker
- Vaping Only
- Unknown/Other
Examples:
1. Input: "Smokes daily, about a pack" → Category: Current Smoker
2. Input: "Quit 2 years ago" → Category: Former Smoker
3. Input: "Never smoked" → Category: Never Smoked
4. Input: "Uses e-cigarettes occasionally" → Category: Vaping Only
5. Input: "No clear response" → Category: Unknown/Other
Now classify:
Input: "[TOBACCO_TEXT]" → Category:

## S.5 Substance Abuse Classification Prompt

You are a clinical classification assistant. Categorize substance use into:
- No Substance Use
- Current Use
- Past Use
- In Recovery
- At Risk
- Unclear/Other
Examples:
1. Input: "Currently using methamphetamines" → Category: Current Use
2. Input: "Recovering from opioid addiction" → Category: In Recovery
3. Input: "Never used drugs" → Category: No Substance Use
4. Input: "Occasional marijuana use in the past" → Category: Past Use
5. Input: "History of use, unsure if still using" → Category: Unclear/Other
Now classify:
Input: "[SUBSTANCE_TEXT]" → Category:

## S.6 Exercise Classification Prompt

Classify the patient’s physical activity level into:
- Regular Exercise
- Sedentary Lifestyle
- Occasional Activity
- Limited Mobility
- Unclear/Other
Examples:
1. Input: "Walks daily for 30 minutes" → Category: Regular Exercise
2. Input: "No time for exercise" → Category: Sedentary Lifestyle
3. Input: "Exercises once or twice a month" → Category: Occasional Activity
4. Input: "Wheelchair bound" → Category: Limited Mobility
5. Input: "Not specified" → Category: Unclear/Other
Now classify:
Input: "[EXERCISE_TEXT]" → Category:

## S.7 Housing Environment Classification Prompt

You are a clinical assistant classifying a patient’s housing situation:
- Stable Housing
- Unstable Housing
- Homeless
- Transitional Housing
- Lives With Others
- Unclear/Other
Examples:
1. Input: "Has own apartment" → Category: Stable Housing
2. Input: "Living in a shelter" → Category: Homeless
3. Input: "Staying temporarily with friends" → Category: Transitional Housing
4. Input: "Lives with parents" → Category: Lives With Others
5. Input: "No mention of housing" → Category: Unclear/Other
Now classify:
Input: "[HOUSING_TEXT]" → Category:

## S.8 Sexual Orientation Classification Prompt

Classify the patient's sexual orientation into:
- Heterosexual
- Homosexual
- Bisexual
- Other Identity
- Declined to Answer
- Unclear/Unknown
Examples:
1. Input: "Straight" → Category: Heterosexual
2. Input: "Gay man" → Category: Homosexual
3. Input: "Bisexual" → Category: Bisexual
4. Input: "Prefers not to say" → Category: Declined to Answer
5. Input: "Queer" → Category: Other Identity
6. Input: "Not clear from note" → Category: Unclear/Unknown
Now classify:
Input: "[SEXUAL_ORIENTATION_TEXT]" → Category:

## S.9 Patient Risk Analysis Explanation Prompt

You are a medical risk analyst. Write a clear and concise explanation (maximum 200 words) for why this patient is classified as at risk for a mental health emergency return. Use plain, clinically relevant language that is easy to understand.

SHAP values indicate the impact of each feature on the model's risk prediction:

A positive SHAP value means the feature increases the patient's risk.

A negative SHAP value means the feature decreases the patient's risk.

Below are the top 10 features most responsible for this patient's classification:

{features}

Population-level context for these features:

{population_stats}

Your explanation should follow this structure:

Start with a brief statement summarizing the patient's overall risk level and top contributing features.

For each feature, explain how it affects risk using clinical terms (e.g., "frequent visits", "elevated heart rate"), and compare to population values if available.

Do not include SHAP values mid-sentence; instead, include them at the end of each item in parentheses (e.g., SHAP=0.245).

Group features with unclear or missing population data together in one paragraph.

Conclude with a one-sentence summary justifying the patient's overall risk classification based on the data.

Avoid using symbols or technical jargon (e.g., no arrows like ↑ or ↓, no equations). Do not include the patient index.

Analysis (max 200 words):
